# *In vivo* skin optical clearing for improving imaging and light-induced therapy: a review

**DOI:** 10.1117/1.JBO.28.6.060901

**Published:** 2023-06-06

**Authors:** Qing Xia, Dongyu Li, Tingting Yu, Jingtan Zhu, Dan Zhu

**Affiliations:** a Huazhong University of Science and Technology, Britton Chance Center for Biomedical Photonics – MoE Key Laboratory for Biomedical Photonics, Wuhan National Laboratory for Optoelectronics – Advanced Biomedical Imaging Facility, Wuhan, China; b Huazhong University of Science and Technology, School of Optical Electronic Information – Advanced Biomedical Imaging Facility, Wuhan, China

**Keywords:** *in vivo* skin optical clearing, optical imaging, skin lesion, laser treatment

## Abstract

**Significance:**

Skin is the largest organ and also the first barrier of body. Skin diseases are common, and cutaneous microcirculation is relative to various diseases. Researchers attempt to develop novel imaging techniques to obtain the complex structure, components, and functions of skin. Modern optical techniques provide a powerful tool with non-invasiveness, but the imaging performance suffers from the turbid character of skin. *In vivo* skin optical clearing technique has been proposed to reduce tissue scattering and enhance penetration depth of light and became a hot topic of research.

**Aim:**

The aim of this review is to provide a comprehensive overview of recent development of *in vivo* skin optical clearing methods, how *in vivo* skin optical clearing enhances imaging performance, and its applications in study and light therapy of various diseases.

**Approach:**

Based on the references published over the last decade, the important milestones on the mechanism, methods, and its fundamental and clinical applications of *in vivo* skin optical clearing technique are provided.

**Results:**

With the deepening understanding of skin optical clearing mechanisms, efficient *in vivo* skin optical clearing methods were constantly screened out. These methods have been combined with various optical imaging techniques to improve imaging performances and acquire deeper and finer skin-related information. In addition, *in vivo* skin optical clearing technique has been widely applied in assisting study of diseases as well as achieving safe, high-efficiency light-induced therapy.

**Conclusions:**

In the last decade, *in vivo* skin optical clearing technique has developed rapidly and played an important role in skin-related studies.

## 1 Introduction

The skin, composed of the epidermis, dermis, and subcutaneous tissues, is the body’s largest organ.^1^ The outermost stratum corneum comprises multiple layers of inactive keratinocytes held together by a lipid-rich matrix. These cells overlap to effectively prevent intravasation of foreign substances and extravasation of body fluids. The remainder of the epidermis consists of the hyaline, granular, spinous, and basal layers. The dermis contains abundant collagen and elastic fibers that provide toughness and elasticity. It is also rich in blood vessels, lymphatics, and nerves that regulate body temperature, sense external stimuli, and provide immune defense. The skin thus plays a crucial role in maintaining the body’s normal physiological state. When lesions occur within the body, the skin may be affected, resulting in cutaneous structural and functional abnormalities that manifest as various diseases.^2^^,^^3^

Optical imaging techniques have been widely applied in skin imaging due to their noninvasiveness, high resolution, and high contrast. However, skin is a turbid medium with a complex composition and varying refractive indices,^4^^,^^5^ which limits the optical imaging quality.

In 1997, Tuchin’s group proposed a tissue optical clearing technique using chemical agents to render tissues transparent.^6^ This technique was quickly applied to skin and garnered significant attention. Initially, researchers explored rendering skin transparent *ex vivo*,^7^ allowing for the acquisition of cutaneous structural information but not permiting observation of live dynamics. Then, researchers began to apply the optical clearing technique to skin *in vivo* by directly injecting optical clearing agents (OCAs) into the dermis to improve skin transmittance. However, this protocol was found to adversely affect skin vasculature^8^ and even cause skin necrosis in some cases.^9^ In 2010, Zhu’s group successfully achieved *in vivo* skin optical clearing imaging by topically applying polyethylene glycol (PEG)-400 and thiazone and observing the distribution of blood flow in rat dermis with high resolution using laser speckle contrast imaging (LSCI).^10^ Since then, more and more *in vivo* skin OCAs have been discovered. Furthermore, researchers attempted to move the optical clearing method to human skin.^11^^–^^13^ In 2016, Leahy’s group successfully improved the *in vivo* OCT imaging quality in human forearm skin using fructose gel.^14^ The applications and potential of *in vivo* skin optical clearing technique on human subjects were explored, and its significance in clinical diagnosis and treatment was highlighted in the book published in 2022.^15^ The *in vivo* skin optical clearing technique enabled various optical imaging techniques to obtain richer and finer skin-related information and has already been applied in the basic and clinical studies, such as identifying skin lesions,^16^ detecting disease-induced cutaneous dysfunction,^17^ and localizing transdermally delivered drugs.^18^ With the aid of skin optical clearing, safe, high-efficiency, and accurate light-induced therapy can be achieved for purposes, such as oncotherapy,^19^ tattoo removal,^20^ and photocoagulation.^21^^,^^22^

This review first examines the development of skin optical clearing methods before detailing the important role of *in vivo* skin optical clearing techniques in enhancing optical imaging performance on skin, facilitating disease study and laser treatment.

## 2 *In Vivo* Skin Optical Clearing Methods

The skin optical clearing technique has rapidly advanced since its proposal, benefiting from ongoing progress in screening OCAs and deepening understanding of underlying mechanisms. Initially, researchers speculated that OCAs with high osmotic pressure and refractive index could cause an outflow of low-refractive-index water in tissue and penetrate the tissue to partially replace interstitial fluid, increasing the refractive index of the liquid matrix and improving refractive index matching between the liquid matrix and skin scatterers. However, subsequent studies revealed that the optical clearing potential of agents was not necessarily correlated with their refractive index or osmotic pressure but was more correlated to their molecular structure.^23^^,^^24^ Molecules with hydroxyl groups can compete for and occupy key sites on dermal collagen that form hydrogen bonds with water molecules, disrupting the surrounding hydration layer, releasing bound water, and destabilizing the triple helix structure of collagen.^25^ Agents with more hydroxyl groups were thus preferred.^26^ Later, agents with a propensity to form higher hydrogen bond bridge types were predicted to have better optical clearing effects.^27^ Higher bridge types span further across the collagen surface and more severely disrupt collagen–collagen and collagen–water interactions. The molecular coverage of agents on key collagen sites is related not only to the span of hydrogen bridges but also to their molecular sizes. Considering these two factors, a more promising indicator, the molecular effective coverage surface area, was proposed.^28^ These indicators increasingly efficiently predicted the optical clearing potential of chemical agents, enabling the identification of sugars and alcohols with high optical clearing potential.^28^^–^^30^

**Fig. 1:**
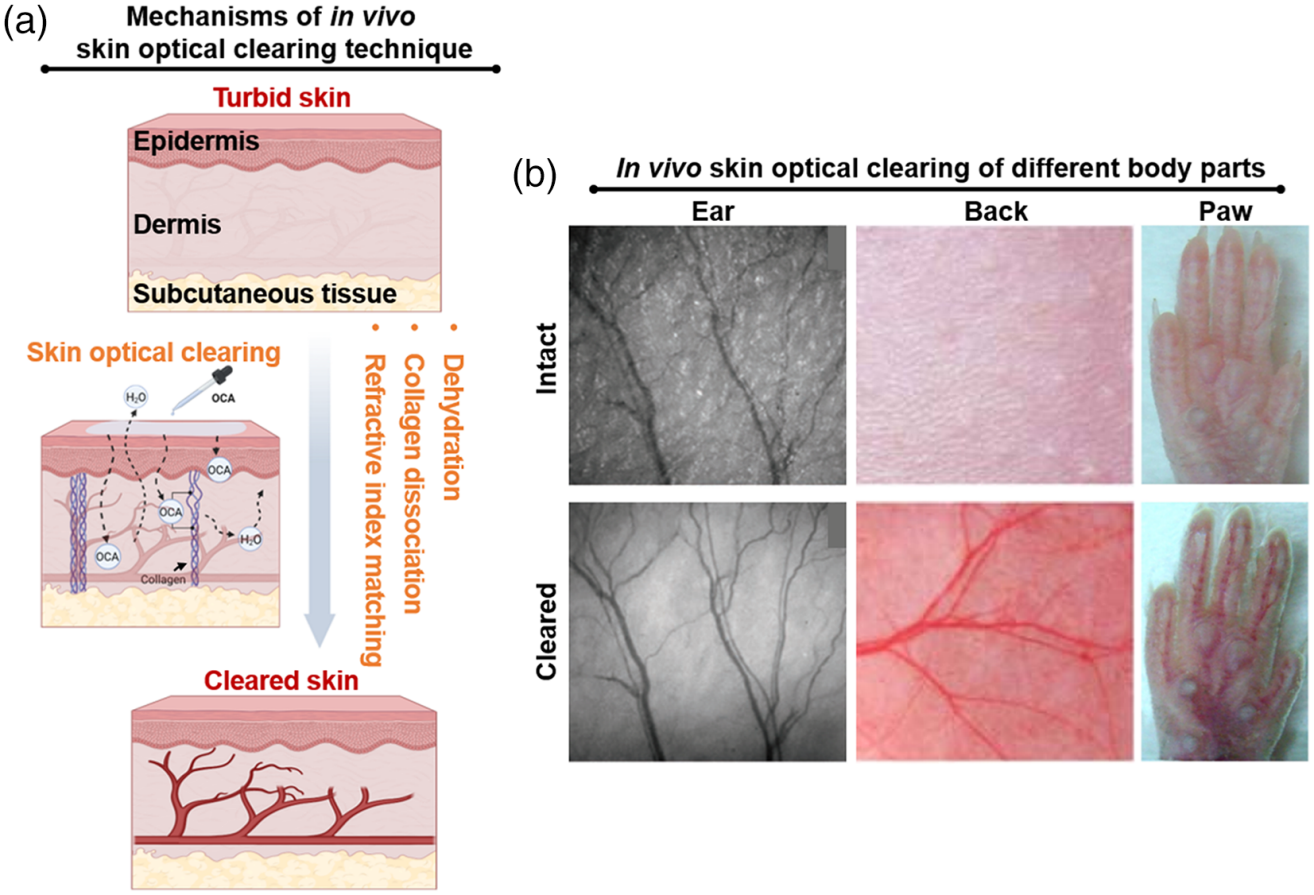
Overview of *in vivo* skin optical clearing technique. (a) Schematic diagram of *in vivo* skin optical clearing technique. The figure was created using BioRender. (b) Typical photographs of skin in different body parts before and after *in vivo* skin optical clearing.^31^^–^^33^

In theory, OCAs can render skin transparent through a combined effect of dehydration, collagen dissociation, and refractive index matching, as shown in Fig. 1(a). However, in realistic *in vivo* applications, the optical clearing effect of agents is sometimes unsatisfactory due to the dense stratum corneum seriously hindering agent penetration into the skin. To overcome this barrier, researchers have proposed various physical methods to promote the penetration of OCAs, including laser irradiation,^13^ fractional laser microablation,^34^ adhesive tape stripping,^35^ sandpaper rubbing,^36^ sonophoresis,^37^ microneedle,^38^ flashlight irradiation,^39^ and more. These methods enhance the OCA penetration by directly removing the stratum corneum and part of the living epidermis, creating microchannels in the skin or generating heat effects to locally disrupt the stratum corneum. Some chemical agents have also been shown to improve the *in vivo* skin optical clearing efficiency to varying degrees, including ethanol,^31^ dimethyl sulfoxide (DMSO),^40^ propylene glycol (PG),^41^^,^^42^ salicylic acid,^43^ liquid paraffin,^11^^,^^44^ hyaluronic acid,^45^ oleic acid,^18^^,^^37^ and so on. As shown in Fig. 1(b), by combining OCAs with penetration-enhancing methods, *in vivo* skin optical clearing methods applicable to different body parts have been successfully developed based on their physiological characteristics.^31^^–^^33^

For *in vivo* skin optical clearing, the biocompatibility of agents is also a major concern. Some clinically used contrast agents with a high refractive index have been proposed as novel OCAs, such as magnetic resonance imaging contrast agent gadobutrol (Gadovist^®^),^46^^,^^47^ gadopentetate dimeglumine (Magnevist^®^),^46^ gadoterate meglumine (Dotarem^®^)^46^ and iso-osmolar x-ray contrast agent iodixanol (Visipaque™),^46^ and iohexol (Omnipaque™).^48^^,^^49^ Their *ex vivo* and (or) *in vivo* applications exhibited a certain optical clearing effect with minor tissue deformation, and no tissue inflammation or hyperemia was observed. It is expected that these contrast agents have high potential for clinical applications.

In the field of optical imaging, diagnosis, and treatment of skin, researchers typically use visible-NIR light and closely monitor changes in tissue reflectance or transmittance in the visible-NIR range when evaluating the effect of skin optical clearing methods.^50^ However, other wavelength bands are also critical for skin-related studies. For instance, moderate ultraviolet exposure can promote the production of vitamin D in the body^51^ and treat skin diseases such as atopic dermatitis and psoriasis.^52^^,^^53^ Terahertz wave can be used to diagnose skin cancer and determine the extent of skin burns based on skin hydration status.^54^ Therefore, researchers have also investigated the applicability of skin OCAs in a wider spectral range. It was verified that *ex vivo* treatment with glycerol could increase the transmittance of colorectal muscle samples at the ultraviolet band (200 to 400 nm) to varying degrees.^55^ The improvement of two bands with central wavelengths located at 230 and 300 nm was particularly significant. In addition, PG and/or glycerol were found to create optical clearing windows in the UV range of gingival mucosa.^56^^–^^58^ For the THz band, the strong absorption of water is a critical factor limiting its propagation in biological tissues. Decreased tissue’s water concentration induced by optical clearing is expected to increase THz transparency.^59^ To identify suitable OCAs for THz applications, Musina et al. comprehensively compared THz-wave absorption and diffusion coefficients in rat brain for multiple agents, including glycerol, PG, DMSO, sucrose, glucose, fructose, dextran 40 and 70, and PEG with different molecular weights. Glycerol was found to be the optimal choice due to its combination of low THz-wave absorption coefficient and high diffusion coefficient.^60^ The ability of glycerol to improve THz imaging was further confirmed in *ex vivo* porcine skin.^61^ The wider spectral application range will greatly broaden the application scenarios of the *in vivo* skin optical clearing technique.

## 3 *In Vivo* Skin Optical Clearing for Improvement of Imaging Performance

Optical imaging techniques have been widely used to obtain structural, component, and functional information about the skin. However, due to the turbid nature of skin, the imaging ability of optical imaging techniques is limited, which can result in missing useful information. To overcome this limitation, researchers have applied the *in vivo* skin optical clearing technique to improve the imaging quality of various optical imaging techniques and explore richer and finer skin-related information.

**Fig. 2:**
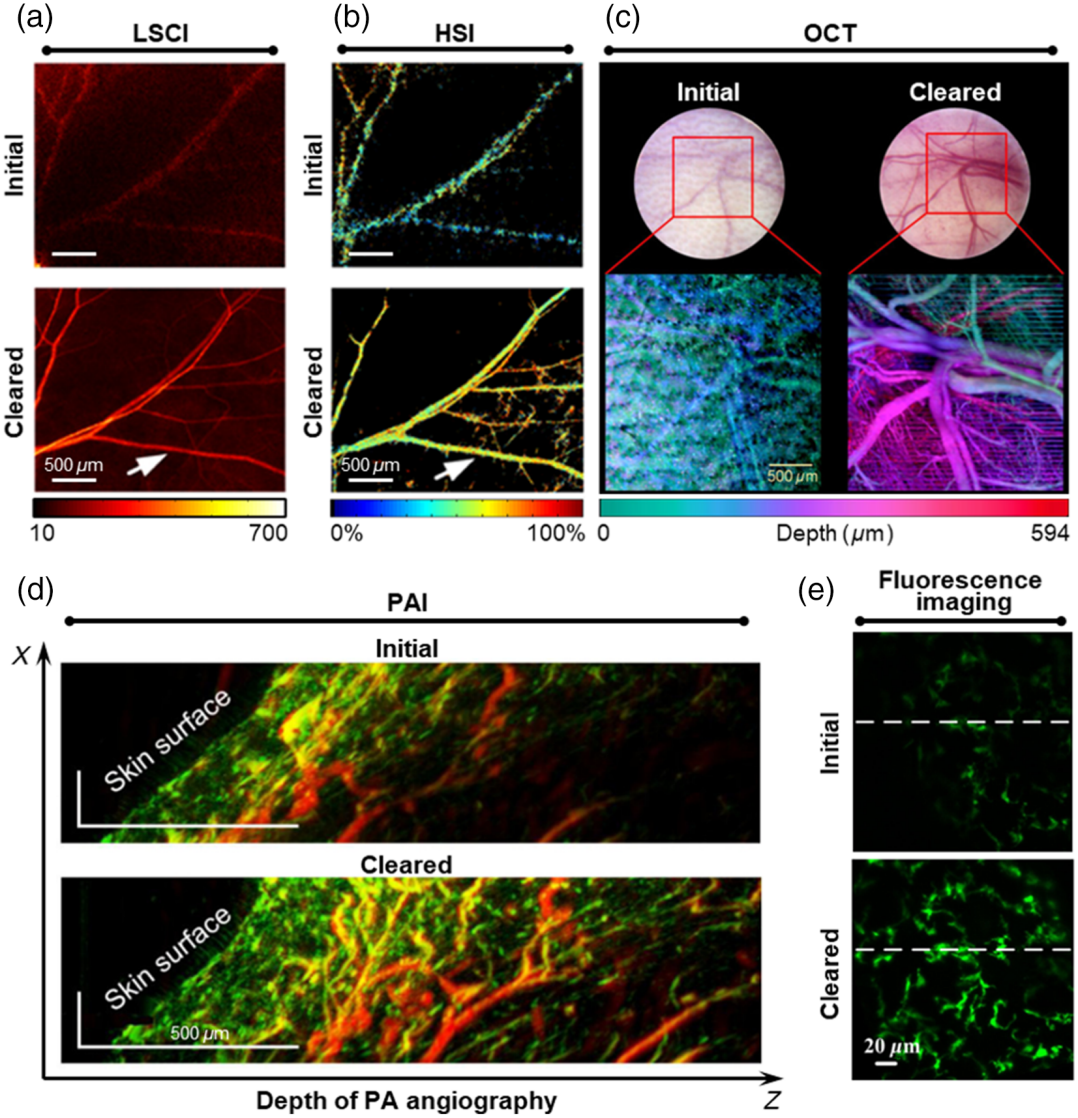
Overview of improvement of optical imaging with *in vivo* skin optical clearing. (a) Blood flow images of mice dorsal skin acquired with LSCI.^62^ (b) Blood oxygen saturation images of mice dorsal skin acquired with HSI.^62^ (c) Projection of angiographic images of mice dorsal skin acquired with OCT.^63^ (d) Angiographic images of mice limb acquired with PAI.^64^ (e) Maximum intensity projection of fluorescence images in mice footpad skin acquired with laser scanning confocal microscopy.^31^

With the assistance of skin optical clearing, LSCI and hyperspectral imaging (HSI) could map cutaneous blood flow and blood oxygen saturation in the mice skin, as shown in Figs. 2(a) and 2(b), enabling the quantitative assessment of hemodynamic responses to stimuli with the single-vessel resolution and improved velocity sensitivity.^62^^,^^65^ Optical coherent tomography (OCT) and its extension could visualize cutaneous layered structures^66^^,^^67^ and detect three-dimensional vascular networks in mice and human skin,^14^^,^^35^^,^^63^ as shown in Fig. 2(c).^35^ Furthermore, photoacoustic imaging (PAI) could provide deeper and richer vasculature in mice limbs when glycerol was mixed with ultrasound gel for acoustic coupling,^64^ as shown in Fig. 2(d). Skin optical clearing also improved Raman spectroscopy to detect stronger and deeper Raman peaks of skin^68^ and assisted surface-enhanced Raman scattering imaging in identifying the shape and boundary of the tumor phantom more accurately,^69^^,^^70^ which would help determine the location of tumors and the presence of metastases. In addition, skin optical clearing increased detection sensitivity of circulating cells by flow cytometry^71^^,^^72^ and improved the signal intensity and imaging depth of laser confocal microscopy when detecting monocytes in mice footpads,^31^ as shown in Fig. 2(e). This advancement would be beneficial for tracking cellular activity deep within the skin.

*In vivo* skin optical clearing technique assists optical imaging to obtain greater imaging depth, higher contrast, resolution, and detection sensitivity, but it should be noted that optical imaging techniques are selective to OCAs. Sometimes OCAs can even have adverse effects on imaging quality. For example, the *in vivo* application of PEG-400 and thiazone on rat dorsal skin could greatly enhance PA amplitude and improve the image quality of deep vessels, whereas DMSO would cause a decrease in PA amplitude, making it a bad choice for improving PAI.^73^ In addition, it is equally important for researchers to focus on the dynamic changes of the optical clearing effect after the application of OCAs. When applied *in vivo* on the skin, agents will penetrate into the skin and cause dehydration. In turn, the skin will react to these changes by rehydration, metabolism of agents, and so on, leading to a reduction in the optical clearing effect. For example, when human palm skin was treated with glucose *in vivo*, the improvement in imaging depth of OCT reached a peak after 15 min and then gradually declined, which indicated that about 15 min after optical clearing was the optimal time to perform imaging in this case.^12^

## 4 *In Vivo* Skin Optical Clearing for Aiding Disease Study

Skin is a common target for both basic and clinical studies. On the one hand, the skin is exposed to the environment and serves as the first line of physiological defense against physical, chemical, and microbial threats, making it particularly susceptible to damage and a variety of associated diseases have not yet been fully understood. On the other hand, there is a close link between the effects of some diseases on the skin and internal organs, such that the skin might be implicated when other organs become diseased. Researchers have applied optical imaging to perform *in vivo* studies of skin lesions,^74^ but acquired limited information. By incorporating *in vivo* skin optical clearing technique, researchers can improve skin transparency and gain better insight into the structures, functions, and components of the skin. This improvement would significantly enhance the understanding of skin conditions under different diseases and underlying mechanisms of skin lesions.

**Fig. 3:**
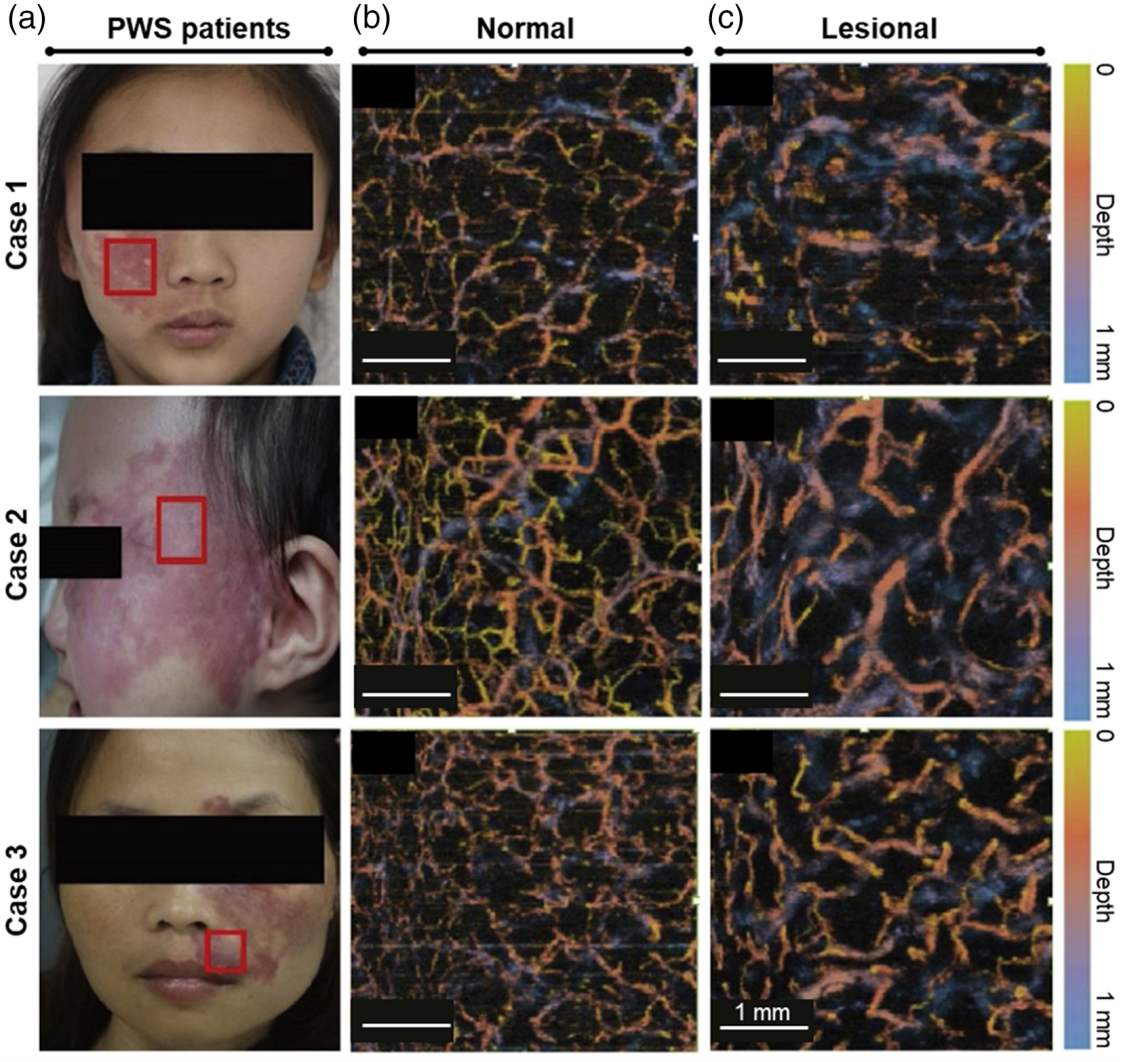
Representative results of a study on PWS assisted with *in vivo* skin optical clearing imaging.^16^ (a) Photographs of PWS patients. The observed lesions of case 1, case 2, and case 3 were located on the cheek, the tempus, and around the lips, respectively. Red boxes represent lesion areas imaged by OCT angiography. Vasculatures of contralateral normal areas (b) and lesion areas (c) of PWS patients captured by OCT angiography after skin optical clearing.

With the assistance of skin optical clearing imaging, the vasculature of facial skin in patients with port-wine stains (PWS) was clearly visualized, and structural parameters of blood vessels at different depths were quantitively evaluated,^16^ as shown in Fig. 3. The vessel density in the superficial layer (<0.21  mm) of the lesion area was lower than that of the contralateral normal skin, while the vessel diameter was similar. However, it was found that the diameter and density of deep blood vessels (>0.21  mm) were significantly higher than that of the contralateral normal skin. These parameters not only reveal the effects of PWS on blood vessels but also help doctors plan treatment. For example, in the laser treatment of PWS based on selective photothermolysis,^75^ setting different laser pulse widths and energies according to the size and involved depth of malformed vessels in the lesion area may achieve better treatment outcomes.

*In vivo* skin optical clearing technique allows continuous monitoring of the dynamic changes in skin and the impact of diseases on it. In the Henoch–Schonlein purpura rat model, changes in the morphology of blood vessels and deposition of immune complexes IgA were recorded *in vivo* during the acute development of skin purpura.^76^ It was found that vascular lesion appeared immediately after the model was established, while the deposition of IgA peaked after 12 h. It was deducted that the vascular lesion enhanced IgA deposition. Similarly, in the psoriatic mice model, cytokines in blood vessels were traced during the development of psoriasis.^77^ A noticeable delay between the emergence of IL-23 and IL-17 was observed, which might help explain the complex interplay among cytokines related to psoriasis.

**Fig. 4:**
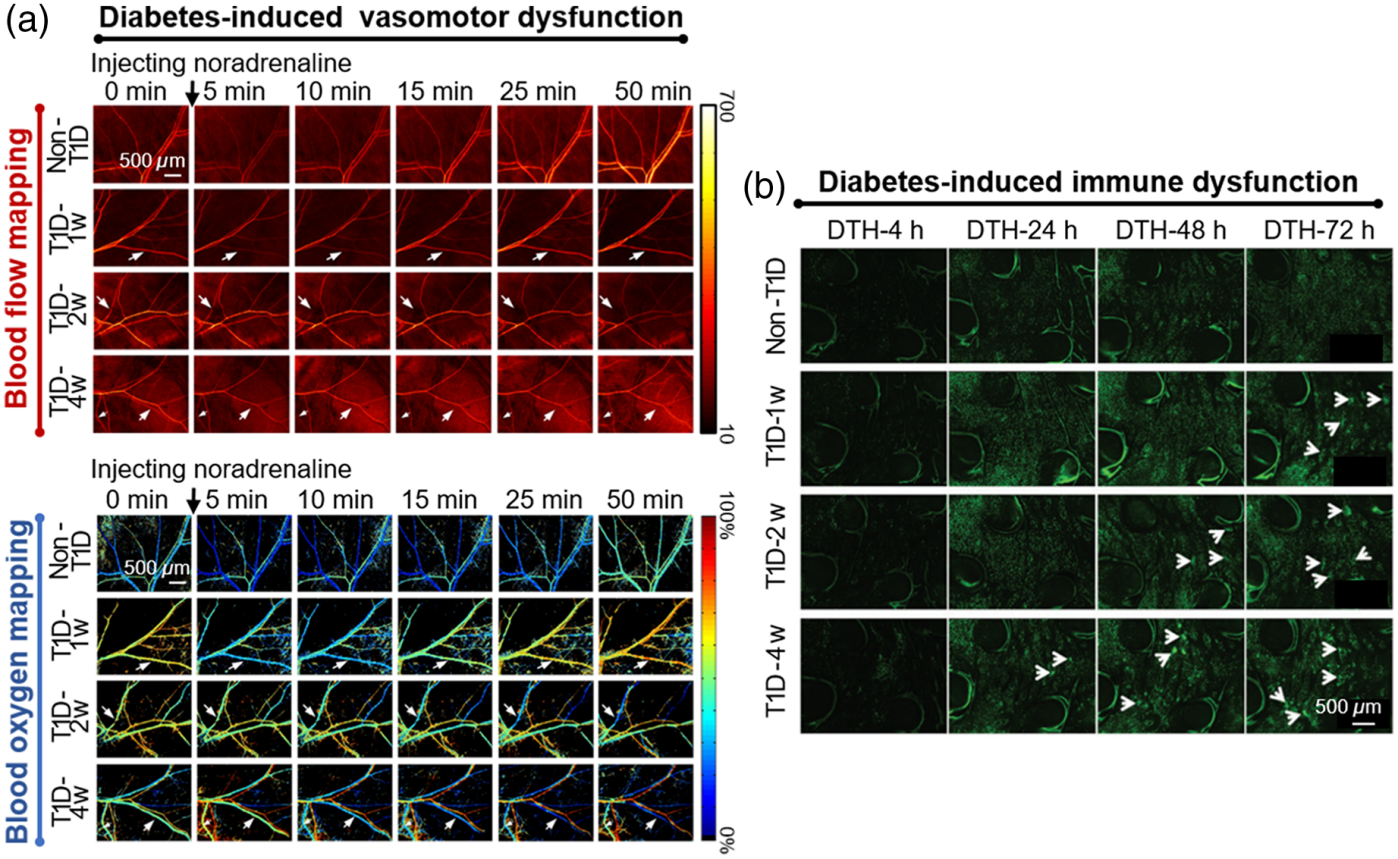
Representative results of basic studies on diabetic-induced cutaneous vascular and immune dysfunction assisted with *in vivo* skin optical clearing imaging. (a) Dynamic responses of blood flow maps and oxygen saturation maps to noradrenaline in non-T1D mice and diabetic mice at different stages (T1D-1 w, T1D-2 w, and T1D-4 w) monitored by LSCI and HSI through an optical clearing skin window.^62^ (b) Recruitment of monocytes/macrophages in mice footpads at different time points (DTH-4 h, DTH-24 h, DTH-48 h, and DTH-72 h) on non-T1D mice and diabetic mice at different stages (T1D-1 w, T1D-2 w, and T1D-4 w) acquired by confocal imaging. White arrows indicate the formed cellular clusters.^79^

Diabetes mellitus is a kind of chronic metabolic disease. Long-term hyperglycemia can cause damage to different organs and tissues^78^ and further induce various complications. Some studies on diabetes-induced skin dysfunction have been conducted with the help of skin optical clearing techniques. To test vasomotor function, the hemodynamic response of mice skin induced by the vasoconstrictor noradrenaline was monitored *in vivo* by LSCI and HSI. The single-vessel resolution achieved by skin optical clearing allowed researchers to analyze abnormal changes in the arteries and veins separately. For normal mice, norepinephrine caused arterial and venous blood flow and blood oxygen levels to decrease within minutes, then return to initial levels or slightly increase within the monitoring time. However, in the mice model of type 1 diabetes (T1D), blood flow recovery became increasingly difficult as T1D progressed, and the decrease of arterial blood oxygen induced by noradrenaline was weakened, as shown in Fig. 4(a). It suggested that diabetes induced vasomotor dysfunction.^62^ Furthermore, the effects of diabetes on cerebral and cutaneous hemodynamic responses to drug stimulation were compared.^80^ Both cerebral and cutaneous blood oxygen responses became abnormal after 1 week, indicating that the cutaneous blood oxygen response has the potential to serve as a good assessment indicator of cerebrovascular dysfunction in the early stage of diabetes. In addition, increased vascular permeability caused by diabetes was directly observed by visualizing the distribution of intravenously injected Evans blue dye in the cutaneous vascular and extravascular regions through a skin optical clearing window. The effect of diabetes on immune function was also studied. Delayed-type hypersensitivity (DTH) was elicited in mice footpads.^79^ Compared with non-T1D mice, the increased infiltration and recruitment of monocytes/macrophages after DTH was found in diabetic mice, as shown in Fig. 4(b), but their motility was significantly attenuated, indicating that the immune function of monocytes/macrophages became abnormal. These tendencies were further potentiated with the progression of diabetes. The improvement of the optical imaging depth with *in vivo* skin optical clearing allowed the comparison of motion behaviors of monocytes/macrophages at different depths around the inflammatory foci. For non-T1D mice, the migration displacement of monocytes/macrophages of the deep dermis was significantly larger than that of the superficial layer in the early stage of DTH (DTH-4 h), but an opposite tendency at the late stage (DTH-72 h), indicating that the cutaneous immune response at different depths was indeed different. Diabetes-induced skin structural change has also been reported. When the tissue structure changes, it will affect the diffusion of agents in the tissue. Therefore, in addition to direct visualization, it is also possible to indirectly diagnose tissue structural changes by measuring the diffusion of agents in the tissues. A comparative study of the diffusion of glucose in *ex vivo* skin samples of normal or alloxan-induced diabetic rats was conducted by measuring dynamic changes of collimated transmittance and geometric parameters (weight, thickness, and area) of samples in the optical clearing process.^81^ It was found that glucose diffusivity was twofold slower for diabetic skin. A similar conclusion was drawn by measuring the diffusion of glycerol in the normal/diabetic skin.^82^ Therefore, monitoring the diffusion of OCAs in the tissues can be used as a reference to distinguish between normal and diseased tissues.

The skin optical clearing technique is not only useful for studying skin lesions but also for detecting substances in the skin. This technique has been shown to enable more complete and deep localization of ${\mathrm{CaCO}}_{3}$ particles in the skin,^18^ which could be valuable for evaluating the penetration effect of novel carrier particles developed for transdermal drug delivery or obtaining pharmacokinetic information about drugs in the skin. In addition, *in vivo* visualization of subcutaneous upconversion nanoparticle microarrays was achieved with high spatial resolution using fluorescence imaging when the skin was cleared. It methodologically demonstrated the potential of skin optical clearing-assisted fluorescence imaging with PDMS microarrays spotted with specific probes for high-throughput detection of disease-related biomarkers in the tissue interstitial fluid.^83^

## 5 *In Vivo* Skin Optical Clearing for Enhancing Light-Induced Therapy

With the advent and rapid development of laser technology, it is becoming increasingly common to use lasers for medical treatment. However, to achieve optimal therapeutic outcomes, the selection of lasers is crucial. Factors, such as laser wavelength, pulse width, and energy, must be carefully considered in accordance with the specific disease, condition, and lesion location.^84^^,^^85^ It is also important to overcome the challenges posed by the tissue above the target area. Scattering and absorption will reduce the luminous flux reaching the target and prevent the light from focusing on the target. While increasing laser power may improve treatment outcomes to a certain extent, it can also lead to greater damage to the surrounding tissues. Simulations have demonstrated that increased skin transparency played a critical role in enhancing the luminous flux reaching the target layer or increasing the proportion of photons absorbed by the target layer, potentially improving treatment efficiency.^20^^,^^86^ Thereby, researchers have tried to introduce the skin optical clearing technique to enhance efficacy of light-induced therapy.

**Fig. 5:**
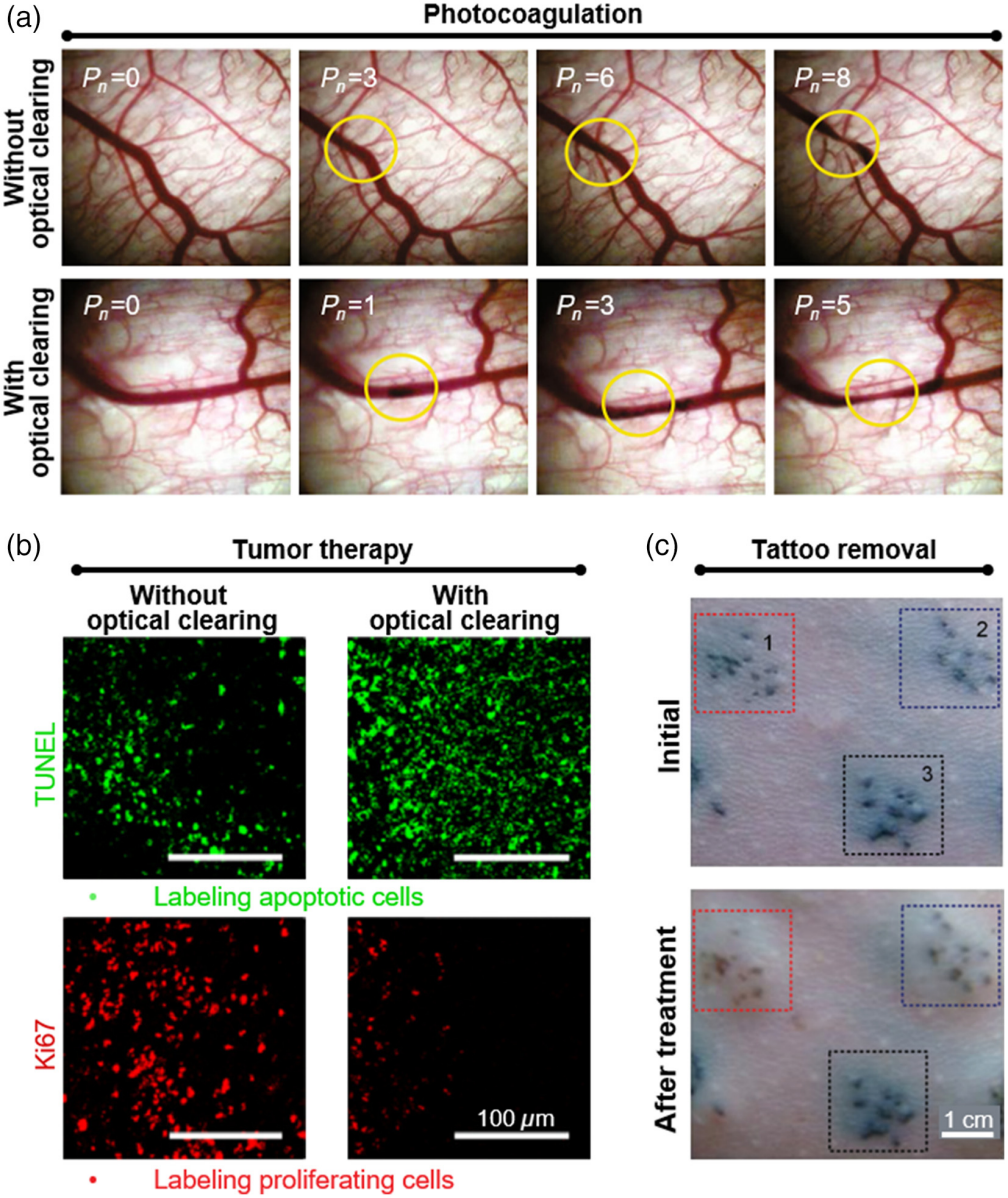
Overview of effects of *in vivo* skin optical clearing on light-induced therapy. (a) Thermal response of blood vessels without or with optical clearing when irradiated with different numbers of laser pulses.^21^ (b) Fluorescence images of TUNEL- and Ki67-labeled tumor slices obtained 14 days after synergistic treatment of photothermo-chemotherapy in a deep-sited tumor model without or with optical clearing.^19^ (c) Photographs of three pigmentation areas on rat dorsal skin before and after different treatments. Area 1: laser irradiation after skin optical clearing; area 2: only laser irradiation; area 3: without treatment.^20^

Skin optical clearing has proven to be a valuable technique in photocoagulation. In the treatment of PWS, multi-pulse Nd: YAG laser was deduced to be powerful by coagulating diseased vessels.^87^^,^^88^ When the OCA was applied to the subcutaneous side of a rat dorsal skin chamber for 10 min, the number of pulses required to achieve the desired treatment endpoint (i.e., thread-like constriction of target blood vessels) was reduced by ∼40% compared with treatments without optical clearing,^21^ as shown in Fig. 5(a). In the photocoagulation of vas deferens for sterilization, an OCA was non-invasively delivered into the scrotal skin around the laser irradiation site, and after 30 min, skin transparency was improved by 26%, which resulted in a reduction in the average power required for laser thermal coagulation of the vas deferens, from 9.2 to 7.0 W.^89^

Combined with skin optical clearing, light-induced therapy, including photodynamic therapy (PDT) and photothermal therapy (PTT), was used to treat deep-sited tumors. An *in vivo* experiment demonstrated that preliminary treatment of OCAs and low-intensity laser irradiation on the skin could reduce skin thermal damage during PTT of subcutaneous tumors.^90^ It not only indicated that light-induced therapy with the aid of skin optical clearing technique produced lower side effects, but also implied that more photons would reach the target tumor, which might lead to higher treatment efficiency. In a pigmented melanoma mouse model, PDT was performed using two commercially available photosensitizers, i.e., photodithazine (PDZ) and Visudyne (VIS).^91^ Dynamic changes in tumor volume were measured after PDT, and it was found that the tumor treated with PDZ-based PDT (PDZ-PDT) kept growing, similar to the untreated group. However, VIS-based PDT (VIS-PDT) and PDT based on both photosensitizers (PDZ-VIS-PDT) could reduce the tumor growth rate, but the tumor volume still increased slowly. When PDT was combined with the skin optical clearing technique, no obvious tumor growth was observed within 8 days after PDZ-PDT, and tumors treated with VIS-PDT and PDZ-VIS-PDT gradually regressed. In another study, a self-developed nano-platform DOX@PGNCs (that is, doxorubicin-loaded gold nanocages) was used to treat tumors with the synergistic effect of photothermo-chemotherapy.^19^ The synergistic therapy was effective in inhibiting growth of superficial tumors, but the effect was greatly weakened in tumors covered by *ex vivo* porcine skin. However, the application of skin OCAs brought a series of positive effects, including increased photothermal conversion efficiency of GNCs, uptake of DOX@PGNCs by tumor cells, and the release of doxorubicin, which promoted tumor cell apoptosis and inhibited tumor cell proliferation to a greater extent, as shown in Fig. 5(b). This greatly slowed the tumor growth rate. In the study of Chu et al., PTT triggered by NIR-II light was performed to enhance the effective depth of *in vivo* cancer therapy, thus inhibiting tumor growth. On this basis, the skin optical clearing technique was further introduced to the therapy, and the complete tumor ablation was well achieved without any reoccurrence.^92^

In addition, skin optical clearing has also been shown to have a positive effect on tattoo removal. When pigmented skin was pretreated with optical clearing, the clearance rate of pigment could reach up to 82% after laser irradiation, compared with only 55% clearance in the pigmented area treated with laser irradiation alone,^20^ as shown in Fig. 5(c). These results suggested that skin optical clearing increased the efficiency of laser tattoo removal by 1.5 times.

Therefore, the application of skin optical clearing can improve treatment efficiency or decrease light dosage, which will greatly enhance the clinical translational potential of light-induced therapy.

## 6 Conclusion and Prospect

Researchers have worked to overcome challenges posed by the strong scattering property of tissues in optical techniques, making significant improvements in optical systems^93^^,^^94^ and image processing.^95^^,^^96^ In recent decades, the novel skin optical clearing technique has been proposed to address this issue at its root by matching the refractive index of skin components. This review primarily covers the development of skin optical clearing methods and their *in vivo* applications in enhancing optical imaging quality, facilitating disease study, and improving the efficiency of laser treatment.

While *in vivo* skin optical clearing has made significant contributions to basic research, its clinical applications remain limited. This is due to the fact that although efficient *in vivo* skin optical windows have been successfully established in experimental animals, human skin is thicker and has a stronger barrier function in the stratum corneum, making *in vivo* optical clearing of human skin less effective than in experimental animals. In the near future, developing high-efficiency and safe *in vivo* human skin optical clearing methods will be of great significance. At the same time, considering differences in human age, skin color, and body parts, niche-targeting skin optical clearing methods should be established for clinical applications, such as pediatric injection, dermoscopy, and ultrasonography.
